# Prognostic value of plasma EGFR ctDNA in NSCLC patients treated with EGFR-TKIs

**DOI:** 10.1371/journal.pone.0173524

**Published:** 2017-03-23

**Authors:** Chengjuan Zhang, Bing Wei, Peng Li, Ke Yang, Zhizhong Wang, Jie Ma, Yongjun Guo

**Affiliations:** 1 Department of Molecular Pathology, Henan Cancer Hospital, The Affiliated Cancer Hospital, Zhengzhou University, Zhengzhou, China; 2 Department of Infectious Diseases, Henan Provincial People's Hospital, Zhengzhou, China; Wayne State University School of Medicine, UNITED STATES

## Abstract

**Objective:**

Epidermal growth factor receptor (EGFR) specific mutations have been known to improve survival of patients with non-small-cell lung carcinoma (NSCLC). However, whether there are any changes of EGFR mutations after targeted therapy and its clinical significance is unclear. This study was to identify the status of EGFR mutations after targeted therapy and predict the prognostic significance for NSCLC patients.

**Methods:**

A total of forty-five (45) NSCLC patients who received EGFR-TKI therapy were enrolled. We identified the changes of EGFR mutations in plasma ctDNA by Amplification Refractory Mutation System (ARMS) PCR technology.

**Results:**

In the 45 cases of NSCLC with EGFR mutations, the EGFR mutation status changed in 26 cases, in which, 12 cases (26.7%) from positive to negative, and 14 cases (31.1%) from T790M mutation negative to positive after TKI targeted therapy. The T790M occurance group had a shorter Progression -Free-Survival (PFS) than the groups of EGFR mutation undetected and EGFR mutation turned out to have no change after EGFR-TKI therapy (p < 0.05).

**Conclusions:**

According to this study, it’s necessary to closely monitor EGFR mutations during follow-up to predict the prognosis of NSCLC patients who are to receive the TKI targeted therapy.

## Introduction

Lung cancer mortality remains to be a serious issue, and is likely to continue to rise globally[1]. Epidermal growth factor receptor (EGFR) specific mutations have been known to be related to the improval of survival in non-small-cell lung carcinoma (NSCLC) patients. Clinically, targeted agents such as gefitinib, erlotinib or afatinib against EGFR have dramatically improved the treatment outcome including Progression-Free-Survival (PFS), Objective Response Rate (ORR) and Overall survival (OS) in patients with specific driver mutations[2,3]. At present, the EGFR- tyrosine kinase inhibitor (TKI) treatment is a standard and first line therapy for NSCLC patients having EGFR-activating mutations[4]. However, acquired resistance often occurs after EGFR- tyrosine kinase inhibitor (TKI) therapy[5,6]. The occurrence of T790M in exon 20 of the EGFR gene is the most common resistance mechanism in NSCLC patients with EGFR-TKI therapy[7,8]. But it remains unclear what’s the EGFR mutations status after TKI therapy and whether only T790M could be detected in these samples. Furthermore, the relationship between the EGFR mutation status with patient clinical outcomes like pathologic features and PFS remains undefined.

In this study, we identified the changes of EGFR mutations in 45 NSCLC patients using AMPS PCR technology. This study showed the different changes of EGFR mutations detected by plasma circulating tumor DNA (ctDNA) after EGFR-TKI targeted therapy which could predict the different prognosis of NSCLC patients.

## Materials and methods

### Patients and samples collection

**Fig 1:**
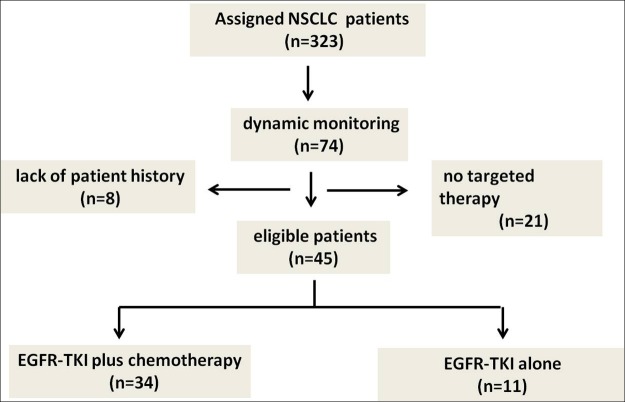
Patient enrollment flow chart. NSCLC, non-small cell lung cancer; EGFR-TKI, epidermal growth factor receptor-tyrosine kinase inhibitor.

In this retrospective study, 323 NSCLC patients collected in Henan Cancer Hospital between 2014 and 2016. EGFR mutations were measured in peripheral blood ctDNA (circulating tumor DNA) of the 323 patients. Among which, 74 (23%) ctDNA samples had paired formalin-fixed paraffin-embedded (FFPE) specimens. EGFR mutations were detected in tumor tissue by the method of ARMS PCR. Twenty-nine (29) samples were excluded because they didn’t have complete medical records or didn’t receive EGFR-TKI (erlotinib, gefitinib or icotinib) targeted therapy. The study was approved by the ethics committee of the Affiliated Cancer Hospital of Zhengzhou University and carried out following the local ethical guidelines. The characteristics of the 45 patients available are shown in Fig 1.

### DNA extraction

NSCLC formalin-fixed paraffin-embedded tissue (FFPE) samples were obtained from primary tumors without any prior targeted therapy, every sample was reviewed and the cell percentage of tumor was estimated by pathologists prior to DNA extraction. Ten (10) FFPE slides were prepared to 5 μm first and deparaffinized in xylene at 56℃ for 10 min, then centrifuged at 13,000 rpm for 2 min, and the supernatant was then discarded. Deparaffinization process was repeated in xylene, and the genomic DNA was extracted with QIAamp FFPE DNA kits (Qiageen, Hilden, Germany) according to the manufacturer’s instructions. Patient blood samples were collected in 5ml tubes containing ethylene diamine tetraacetic acid (EDTA) and were centrifuged at 3,000 rpm for 10 min at 4°C within 2 hours of collection. The plasma supernatant were isolated in 1.5 ml Eppendorf tubes, and centrifuged at 3,000 rpm for 5 min at 4°C. The plasma supernatants then were transferred to new 1.5 ml Eppendorf tubes, and DNA was extracted with QIAamp Circulating Nucleic Acid kits (Qiagen, Hilden, Germany) according to the manufacturer’s instructions. DNA quality and quantity were assessed on Thermo Scientific NANODROP 2000.

### Detection of EGFR mutations by AMPS PCR

EGFR mutations were detected using AMPS PCR technology. AMPS PCR was performed using the Human EGFR Gene Mutation Quantitative Detection Kit (Fluorescent PCR) (Beijing ACCB Biotech Ltd, China) which had passed ISO13485 quality management system and obtained CE certification and CFDA registration on an Agilent Technologies Stratagene Mx3000P Real-Time PCR system following manufacturer instructions. This kit usually requires 150 ng extracted DNA being able to detect 45 known recurrent mutations in EGFR exons 18–21, which include G719 mutations (including G719S, G719C and G719A) in exon 18, 31 deletions (including E746_A750del 2235_2249del15, E746_A750del 2236_2250del15, E746_T751>A, E746_T751>I, L747_T751del 2238_2252del15, L747_T751del 2239_2253del15, L747_T751del 2240_2254del15, K745_E749del, E746_T751>A, L747_P753>S, L747_S752del, E746_S752>D, L747_P753>Q, E746_T751del 2236_2253del18, E746_S752>A, E746_S752>V 2237_2252>T, E746_T751del 2235_2252del18, E746_S752>I, E746_S752>V 2237_2256>TC, L747_A750>P, L747_E749del, L747_A750>P, E746_A750>QP, E746_A750>RP, E746_A750>IP, L747_T751>P, L747_T751>S, L747_T751>Q 2238_2252>GCA, L747_T751>Q 2239_2252>CA, E746_E749del, E746_A751>IP), 3 point mutations (including L858R 2573T>G, L858R 2573_2574TG>GT, and L861Q in exon 21, 2 point mutations (including T790M and S768I and 6 insertion mutations (including V769_D770insASV 2307_2308insGCCAGCGTG, V769_D770insASV 2309_2310AC> CCAGCGTGGAT, D770_N771insSVD, D770_N771insG, H773_V774insNPH, H773_V774insH) in exon 20. The thermal cycling started with 95℃ for 10 min, followed by 40 cycles of 95℃ for 15 s and 60℃ for 60 s, and fluorescent signals were collected at the each end of 60℃ 60 s. As confirmed in our routine clinical application and the manufacturer indication, the assay was capable of detecting EGFR mutations at a frequency of approximate 1% with the lowest DNA concentration 5 ng/μl.

### Detection of EGFR mutations by droplet digital PCR (ddPCR)

Droplet Digital PCR (ddPCR) was performed using the Human EGFR Gene Mutation Detection Kit (ddPCR) (Shanghai source Biological Medicine Technology Co., Ltd, China). First of all, the droplets need to be prepared using QX200 generator (Bio-Rad Laboratories, Inc., Hercules, CA, USA) and were sealed with a preheated PX1 thermal sealing apparatus (180℃ for 5s) after transferred to the 96-well plate. The PCR amplification started with 95℃ for 10 min, followed by 40 cycles of 94℃ for 15 s and 58℃ for 60 s, and then 98℃ for 10 min and 4℃ for 5 min (C1000, Bio-Rad). After the PCR amplification and fluorescently labeled, the droplets were read in an automated droplet flow cytometer (QX200 reader, Bio-Rad) and were analysed using QuantaSoft (Bio-Rad) software.

### Statistical analysis

McNemar test was used to compare the EGFR mutations status before and after EGFR-TKI therapy. The PFS analysis of the different group was performed using the Kaplan-Meier method. Statistically significant difference in the three groups was then defined by a log-rank test (Mantel Cox, 95% CI) of the 3 Kaplan-Meier curves. Differences were considered statistically significant when the *p* value was 0.05 or less. All statistical tests were two-sided.

## Results

### Patients characteristics

The characteristics of the 45 NSCLC patients analyzed in the study are shown in Table 1. There were 13 men and 32 women. 71.1% patients at diagnosis were in the age range of 45–65 years old. Three patients (6.7%) had squamous cell carcinoma and 42 (93.3%) had adenocarcinoma. And the left site was the pathologic feature of 45 NSCLC patients who had the chemotherapy account for 75.6% and radiotherapy account for 35.6%. The data demonstrated that the patient characteristics had no obvious difference between undetected, no change and T790M occurrence groups (*p* > 0.05).

**Table 1:** Patient Characteristics.

	No. of Patients (n = 45)	Percent (%)	χ^2^	P
**Gender**			2.204	0.332
Males	13	28.9		
Females	32	71.1		
**Age(yr)**			2.886	0.089
≤45	6	13.3		
45~65	32	71.1		
>65	7	15.6		
Pathological pattern*			1.456	0.746
Squamous carcinoma	3	6.7		
**Adenocarcinoma**	42	93.3		
Sites*			9.973	0.738
→	11	24.4		
↗	11	24.4		
↘	2	4.4		
←	12	26.7		
↖	3	6.7		
↙	6	13.3		
**Chemotherapy**			2.217	0.330
Yes	34	75.6		
No	11	24.4		
**Radiotherapy**			3.278	0.194
Yes	16	35.6		
No	29	64.4		

*analyzed by Fisher‘s exact test.

“→” shows the tumor is located on the right side of the lung, “↗”shows the tumor is located on the right upper side of the lung, “↘”shows the tumor is located on the right lower side of the lung, “←”shows the tumor is located on the left side of the lung, “↖”shows the tumor is located on the left upper of the lung, “↙”shows the tumor is located on the left lower of the lung.

### Comparative analysis of different detection methods

We choose 35 cases to detect EGFR mutations through ddPCR, which had high sensitivity, strong specificity and can be absolutely quantitative for the detection of EGFR mutations. Results showed that the EGFR mutations were totally the same (100% consistence) after comparing the two different methods ARMS PCR and ddPCR). Therefore, the detection of the plasma EGFR ctDNA through the ARMS PCR technology appears to be a highly sensitive method.

### The influence of chemotherapy on EGFR mutations status

EGFR mutations results obtained by ARMS PCR in the 45 assessable primary NSCLC patients before and after chemotherapy are shown in Table 2. Twenty-five (25) of them (73.5%) had EGFR mutations both before and after chemotherapy, one (1) of them (2.9%) did not have EGFR mutations before and after chemotherapy (McNemar test, c2 = 2.500, v = 1, P > 0.05). The data shows that the status of EGFR mutations were almost not affected by chemotherapy.

**Table 2:** The influence of chemotherapy on EGFR mutations status.

	Before		
	+	-	Total
After			
**+**	**25**	**0**	**25**
**-**	**8**	**1**	**9**
**Total**	**33**	**1**	**34**

Before: The cases of EGFR mutation before chemotherapy. After: The cases of EGFR mutation after chemotherapy.

### The influence of EGFR-TKI targeted therapy on EGFR mutation status

EGFR mutations were obtained by ARMS PCR in the 45 assessable primary NSCLC patients before and after TKI therapy, and are shown in Table 3. There were 32 of the cases (71.1%) with EGFR mutation both before and after TKI therapy. Twelve (12) of the cases (26.7%) with EGFR mutations before TKI therapy turned out to be EGFR mutation negative after TKI therapy (McNemar test, c2 = 6.667, v = 1, P<0.05) (Table 3). Nineteen (19) of the cases (42.2%) with EGFR mutations before TKI therapy turned out to be with no change after TKI therapy, and 14 of the cases (31.1%) had EGFR mutation before TKI therapy and had T790M occurrence after TKI therapy (Table 4). the 3 different groups, including undetectable mutations after, no changes and T790M occurrences after EGFR-TKI therapy had obviously different prognosis with 14.7, 12.2 and 8.4 months prior to change respectively. The data showed that EGFR mutation status have impact on the results of TKI therapy.

**Table 3:** The influence of TKI therapy on EGFR mutations status.

	Before		
	+	-	Total
After			
**+**	**32**	**0**	**32**
**-**	**12**	**1**	**13**
**Total**	**44**	**1**	**45**

Before: The cases of EGFR mutation before TKI therapy. After: The cases of EGFR mutation after TKI therapy.

**Table 4:** The changes of EGFR mutations after TKI targeted therapy.

	Cases	Percent (%)	Median (months)
**Undetected**	**12**	**26.7**	**14.7**
**No change**	**19**	**42.2**	**12.2**
**T790M occurrence**	**14**	**31.1**	**8.4**

### Comparison of progress-free survival (PFS) of patients with different changes of EGFR mutation status after TKI targeted therapy

**Fig 2:**
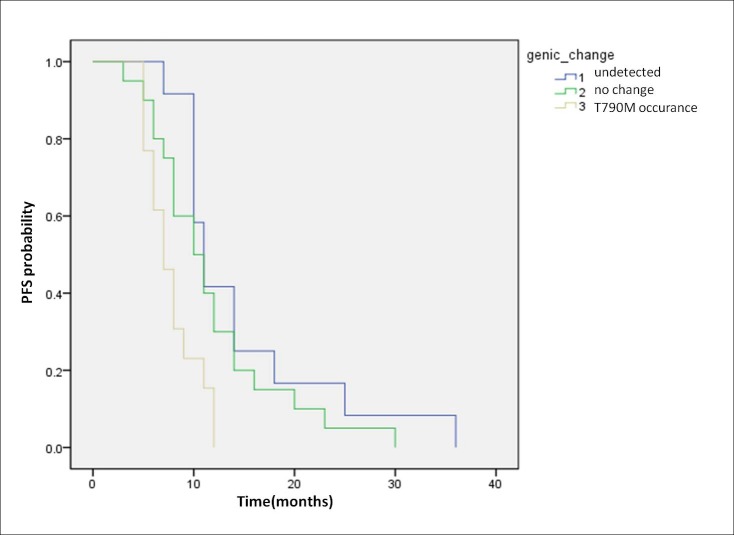
Kaplan-Meier curve stratified by PFS in 45 patients treated with EGFR-TKI.

Among the 45 patients who received EGFR-TKI treatment, PFS was evaluated to compare the prognosis among the different groups. As we can see in Fig 2, the group with EGFR mutation undetected after EGFR-TKI therapy had a longer PFS compared to the T790M occurrence group after EGFR-TKI (*p* < 0.01) (Table 5), and the group with EGFR mutation turned out to have no change after EGFR-TKI also had a longer PFS compared to the T790M occurrence group after EGFR-TKI (*p* < 0.05) (Table 5).

**Table 5:** The results of paired comparison with different groups in 45 patients treated with EGFR-TKI.

	genic_change	1		2		3	
		χ^2^	p	χ^2^	p	χ^2^	p
**Log Rank (Mantel-Cox)**	**1**			**1.176**	**.278**	**9.632**	**.002**
	**2**	**1.176**	**.278**			**5.273**	**.022**
	**3**	**9.632**	**.002**	**5.273**	**.022**		

## Discussion

Targeted therapy has brought new hope to patients with advanced lung cancer both in the prolongation of the survival time and the improvement of the quality of life. However, drug resistance is a problem that we have to face when receiving targeted therapy[9]. Drug resistance has become the main obstacle to the treatment of cancer, and it has drawn extensive research[10]. Clinically, advanced NSCLC patients usually have progression of disease due to acquired drug resistance after 9 to 13 months treatment with EGFR-TKI, and it’s a concerned problem on how to treat after drug resistance[11–13], and many related clinical trials were reported[14,15]. JC Soria and YL Wu found that NSCLC patients who acquired drug resistance when receiving targeted therapy, were not suitable for the combination of targeted drugs and chemotherapy. They can use chemotherapy only, because the combination of the two drugs has no help in prolonging the survival time of the patients[16].Therefore, dynamic monitoring is very important to discover drug resistance timely.

Currently, ARMS PCR has become one of the most important and advanced technologies in the molecular detection of cancer individuals, and its advantages in clinical applications have been widely recognized in industry[17,18]. The detection sensitivity of ARMS method was significantly higher than that of other methods, such as direct sequencing method, which can detect the mutation of 0.1–1.0% gene in samples. In the present study, we demonstrated the changes of EGFR mutation of 45 NSCLC patients through AMPS PCR technology with tumor tissues, and monitored the EGFR mutation status by peripheral blood ctDNA. We also compared the plasma EGFR ctDNA With ddPCR(droplet digital PCR) in 35 cases. Results is not shown here but showed that the EGFR mutations of all the samples were highly consistent (100% consistence). Therefore, the detection of the plasma EGFR ctDNA through the ARMS PCR technology appears to be a highly sensitive method. At the same time, as a non-invasive liquid biopsy, the ctDNA in plasma has been proposed to detect genetic alterations without additional burden and risk to patients[19–21], which also can provide information on molecular evolution of the tumors.

T790M gene mutation accounted for around 50% of all mutations when of the target therapy resistance occurs[22]. The T790M mutation could cause the changes of EGFR space conception and increase the affinity for ATP, which eventually leads to weaker binding of the EGFR-TK region to TKI[23,24]. Our study showed that in the 45 patients receiving the EGFR-TKI treatment, the three different groups of molecular signatures (mutation negativity, no mutations and T790M occurrence) after EGFR-TKI therapy, had obviously different prognostic survival rate at 14.7, 12.2 and 8.4 months prior to change, respectively. The T790M mutation group had the shortest PFS, and needs to change the drug to AZD9291 (AstraZeneca, Macclesfield, UK), which was an irreversible, selective compound that can target the T790M resistance mutation[25–27]. There is no statistical significance between the mutation-negative group and the group with no change after EGFR-TKI therapy (p = 0.278), but the mutation-negative group had longer median PFS than the group with no change after EGFR-TKI therapy. Thus, our data showed that EGFR mutation status affects the results of TKI therapy.

Because of the heterogeneity of tumor, there are individual-dependent different resistance mechanisms, same patient may also has multiple drug resistance mechanisms. Therefore, it’s very important for patients to receive noninvasive and accurate identification of the mechanism behind target therapy resistance. The detection of plasma ctDNA is an effective method for the dynamic detection of gene mutations[28–30]. However, mutations in the T790M gene account only for 50% of all resistance mutations. Mutations also involves other genes and gene amplification[31,32]. Therefore, we may need more monitorings in order to detect resistance timely. For example, we can detect the gene status of MET amplification and K-ras mutations and check CEA (carcinoembryonic antigen) levels periodically, and the others, so as to provide more references to the physicians.

In conclusion, we have demonstrated the feasibility of monitoring EGFR mutation dynamics on NSCLC patients receiving TKI therapy to predict treatment effect. Our results highlight the clinical utility of monitoring EGFR mutations in guiding TKI therapies for NSCLC patients, and in providing important other information for patients’ treatment and prognosis.

## Supporting information

S1 Fig CT-DNA data. (XLSX)
